# A Comprehensive Review Study on Glomerulonephritis Associated With Post-streptococcal Infection

**DOI:** 10.7759/cureus.20212

**Published:** 2021-12-06

**Authors:** Mustafa A Alhamoud, Ibrahim Z Salloot, Shamim S Mohiuddin, Turki M AlHarbi, Faisal Batouq, Naif Y Alfrayyan, Ahmad I Alhashem, Mohammad Alaskar

**Affiliations:** 1 Medicine, College of Medicine, King Fahd Hospital of the University, Imam Abdulrahman Bin Faisal University, Dammam, SAU; 2 Biochemistry, College of Medicine, King Fahd Hospital of the University, Imam Abdulrahman Bin Faisal University, Dammam, SAU

**Keywords:** post infection, glomerulonephritis, gas, psgn, : post streptococcal glomerulonephritis

## Abstract

Post-streptococcal glomerulonephritis (PSGN) is an immune-complex mediated inflammation that used to be considered one of the commonest causes of acute nephritis amongst children. PSGN is characterized by the proliferation of cellular elements called nephritogenic M type as a result of an immunologic mechanism following an infection of the skin (impetigo) or throat (pharyngitis) caused by nephritogenic strains of group A beta-hemolytic streptococci, a gram-positive bacteria that enters the body across pores in the skin or mucus epithelia and is responsible for more than 500,000 deaths annually due to multiple subsequence diseases such as rheumatic heart disease, rheumatic fever, PSGN, and other invasive infections. After the infection, the formation of an immune complex of antigen-antibody and complement system will take place and will deposit in the glomeruli where the injury occurs and leads to inflammation. The manifestations of PSGN can be explained by nephritic syndrome manifestation. PSGN is diagnosed by laboratory tests like microscopy and urinalysis. The imaging studies in PSGN could be used to assess the possible complications of PSGN such as pulmonary congestion and chronic kidney disease. The management of PSGN is symptomatic. If PSGN is not treated, the patient may develop chronic kidney disease. The main way to prevent PSGN is to treat group A streptococcal (GAS) infections by giving good coverage of antibiotic therapy to a patient who has primary GAS infections to prevent the development of the complication.

## Introduction and background

Post-streptococcal glomerulonephritis (PSGN) is a disease that occurs due to an untreated infection with distinct nephritogenic strains of the A beta-hemolytic streptococcal family. PSGN is perceived to be one of the most common causes of acute nephritis in children worldwide. Out of 470,000 cases globally, 97% were found in third-world countries [1]. Clinical presentation varies amongst patients with PSGN. They may be asymptomatic, they can present with microscopic hematuria or with a full-scale nephritic syndrome. Nephritic syndrome is characterized by red or brownish urine due to the existence of excess protein (proteinuria). Proteinuria, if it is within the nephritic range, can cause edema, hypertension, and acute kidney injury. The prognosis of PSGN is often excellent, especially amongst children. In some rare cases, the long-term outcomes may not be benign. PSGN is an immune-complex mediated inflammation of the glomerulus which is a collection of capillaries in the kidney's functional unit the nephron and causes acute nephritic syndrome. PSGN is manifested by a proliferation of cellular elements secondary to an immunologic mechanism after an infection of the skin (impetigo) or throat (pharyngitis) caused by nephritogenic strains of group A beta-hemolytic streptococci, which is a gram-positive bacteria that invades the body through pores in the skin or mucus epithelia. It causes more than 500,000 deaths per year due to multiple diseases such as rheumatic heart disease, rheumatic fever, PSGN, and other invasive infections.

## Review

Global incidence and prevalence of PSGN

Burden of PSGN Around the World and the History of the Disease

Post-streptococcal glomerulonephritis is one of the most common causes of acute nephritis in children worldwide. Moreover, it has a high prevalence in developing countries. The estimated yearly cases of PSGN worldwide is 472,000, more than 95% of cases are in countries with low socioeconomic rank. Streptococcal infection is treated promptly [1]. Easy accessibility to the treatment of streptococcal infections is believed to be the reason behind the decreased incidence of PSGN, especially in developed countries. The risk increases in elders and children between five to 12 years with an annual incidence that ranges from 9.5 to 28.5 per 100,000 individuals. The incidence of PSGN in children infected is approximately 5 to 10% with pharyngitis and 25% with skin infections and studies have shown that it is more frequent in males. PSGN is now a disease that affects the elderly (ages 60 and above) who suffer from devitalizing conditions like alcoholism, malignancies, or diabetes. PSGN is perceived to be a sporadic case. During an epidemic of group A streptococcal (GAS) infection (skin and throat infections), the incidence of PSGN estimated in such cases amongst children is presumed to be 25% with skin infections and 5 to 10% with pharyngitis [1,2].

Epidemics, Incidences, and Prevalence of Acute PSGN Worldwide

**Figure 1:**
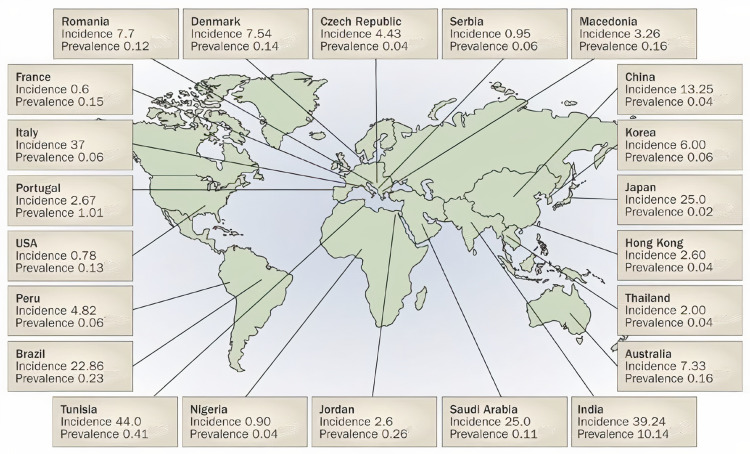
Estimates of post-streptococcal glomerulonephritis (PSGN) prevalence and incidence in 22 countries.

Acute PSGN has shown as an epidemic in mostly rural populations. Table 1 shows the epidemics from 1952 to 2005 that have been reported worldwide. For example, Kuwait has reported an epidemic from 1980 to 1989 with 234 cases mostly after throat or skin infection [3,4]. Figure 1 illustrates the incidence and prevalence of PSGN in 22 countries [5]. In Saudi Arabia, the incidence of PSGN is 25 and the prevalence is 0.11.

**Table 1:** Acute post-streptococcal glomerulonephritis (PSGN) epidemic with clusters worldwide.

Year	Location	Population	Site of Infection	No. of Cases	Streptococcal Type
1952	Nova Scotia, Canada	Rural	Throat	22	ND
1953	Red Lake, MN	Aboriginal	Skin	63	M49
1960	Memphis, TN	Urban	Skin	57	M1, M12, M49
1966	Red Lake, MN	Aboriginal	Skin	27	M49
1975 to 1977	Alaska	Eskimo Children	Skin	75	ND
1978 to 1982	Santiago, Chile	Urban	Skin and Throat	84	ND
1978 to 1983	New Zealand	Urban and Rural	Skin	Clusters (autumn)	M49, 57, 60
1980	Las Tunas, Cuba	Rural	Skin	12	M49, M12
1980 to 1989	Kuwait	Urban and Rural	Skin and Throat	234 cases in 9 yr	M12, M18, M49
1980 to 1998	North Territory, Australia	Rural	Skin and Throat	Clusters	ND
1982 to 1993	Belgrade, Yugoslavia	Military academy	Throat	Clusters of 6 to 24 cases	ND
1883	North Yorkshire, UK	Rural	Unpasteurized Milk	Clusters	S. zooepidemicus
1992	Saga, Japan	Rural	Throat	42	M1
1993	North Queensland, Australia	Aborigines	Skin	58	ND
1993	Brisbane, Australia	Rural	Unpasteurized Milk	Clusters	S. zooepidemicus
2005	Linkoping, Sweden	Rural	Throat	Clusters	S. pyogenes S. constellatus

Causes and risk factors of PSGN

As the name indicates, PSGN is caused by group A streptococcus of nephritogenic M type. Due to pharyngitis, the most common nephritogenic types are 12, 4, 1, 3 25, and 49, and in PSGN following skin infection like impetigo, usually occurs with types 49, 55, 2, 57, or 60. These bacteria also have beta-hemolytic activity because of an enzyme called streptolysin that causes hemolysis, which means lysis or destruction in red blood cells (RBC). It mostly occurs in elderly people over 60 years old and children between five and 12 years old. It is scarcely found in developed countries [6].

Untreated GAS Infection

It starts as acute nephritis, which appears after one to three weeks of untreated streptococcal infection, such as throat infection, otitis media, or cellulitis. The latent period between the development of symptoms of renal involvement and the infection represents the time taken for the formation of immune complex, deposition to the glomeruli where the injury occurs "inflammation" and is dependent upon the site of infection: between one to three weeks following GAS pharyngitis and between three to six weeks following GAS skin infection.

Predisposing Factors

There are some diseases that predispose people to PSGN such as systemic inflammatory disorders like vasculatures (systemic lupus erythematosus [SLE], rheumatoid arthritis, scleroderma, hemolytic uremic syndrome, and cryoglobulinemia), metabolic disorders like thyroiditis and diabetes mellitus, deposition diseases such as amyloidosis, or hereditary disorders like basement membrane disease, and nail-patella syndrome.

Pathophysiology of PSGN

PSGN is caused by glomerular immune-complex disease that is caused by a group of bacteria called group A streptococci. These bacteria have a hemolytic activity by secreting an enzyme called streptolysin. The enzyme lysis the red blood cells and cause complete lysis of the cell which is called beta-hemolytic activity. Also, the bacteria have nephritogenic strains which have a virulence factor that causes the pathogenicity called M protein virulence factor, which permits the bacteria to counter and make the bacteria pass through the host barriers and defenses [7]. The infection by group A beta-hemolytic streptococcus bacteria (GABS) induces a Type lll hypersensitivity reaction, which is characterized with antigen-antibody immune-complex formation, where the antigen from the GAS bacteria binds with antibodies, usually IgG or IgM. After that, the immune complex is carried by the blood to the glomerulus where it gets trapped. Usually, the immune complexes are trapped in the glomeruli in the glomerular basement membrane (GBM). Moreover, it may become sub-endothelial in podocytes, which are epithelial cells. This occurs when the bacterial antigens are initially trapped in the glomeruli and then the antibodies bind with the antigens inside the glomerulus itself [8]. After the deposition in the glomeruli, the immune complex causes inflammation and activation with deposition of C3 complement, oxidant, proteases, and inflammatory cytokines. The inflammation will damage the glomerulus, and eventually cause an impermeability of the glomerulus wall, that will allow larger molecules to move through or filter into the nephron. After losing the permeability of the glomerulus, larger molecules like red blood cells will filter inside and appear in the urine. Moreover, the protein could move through the glomerulus and accumulate in the urine. Therefore, the urine will take on a cola-like color. Furthermore, continuous deposition of immune-mediated complexes will lead to mesangial cell proliferation and leukocyte infiltration resulting in a reduction of the glomerular filtration rate (GFR). Reduced GFR may lead to disturbance of renal functions manifested as electrolyte abnormalities and acid-base imbalance. Moreover, patients may develop nephritic syndrome features such as hypertension and hypervolemia eventually leading to oliguria or anuria (renal failure) [9,10].

Signs and symptoms of PSGN

The signs and symptoms can be deceptive at the beginning of the first infection because they are divided into two stages: the earlier symptoms stage and the latent stage (most patients are asymptomatic but they can develop signs that are manifested clinically). The symptoms of PSGN start to appear after one to three or one to six weeks. (Within this time, they're not the symptoms of PSGN, they're the symptoms of the first infection of GAS, depending on the site of infection like throat, skin, ears, etc., That is why there is a variation in symptoms that start to appear.) In other words, the patient must have a history of GAS infection in order for PSGN to be suspected [11,12]. The initial symptoms are the symptoms of an infection of GAS in other organs or systems in the body such as throat, ears, or skin [11,12]. Earlier symptoms before three to six weeks are sore throat, swollen tonsils covered in a white coating, fever, headache, skin rash, and otitis media. Latent stage "signs" (PSGN manifestations) after three to six weeks are peripheral edema, water retention, hypertension, and nephritic syndrome (detected by urine examination). Once the body produces antibodies and they migrate to the glomeruli that are infected with the bacteria, the inflammation starts because of the immune complex and the inflammatory mediators. It then undergoes a syndrome called nephritic syndrome which consists of hematuria, proteinuria, and oliguria [13,14]. The nephritic syndrome is when the glomeruli get inflamed and the function of filtration is impaired because the epithelial cell (podocyte) is damaged, as a result large molecules such as RBCs (hematuria) and proteins (proteinuria) start to filter through it thus causing it to appear cola-like in color. As a complication, urine production from the kidneys will be decreased (oliguria or anuria). Furthermore, because of salt and water retention, patients will develop hypertension, peripheral edema, and in severe cases, respiratory distress might occur secondary to pulmonary edema [11-14].

Diagnosis of PSGN

Laboratory and Imaging Tests

PSGN is clinically diagnosed through multiple lab and imaging tests, in which each test has a positive correlation to the existence of PSGN. The laboratory tests are microscopic, urinalysis, blood samples, and serology. The imaging studies are ultrasounds and X-rays [10]. In a renal biopsy, the purpose is not mainly to diagnose but rather to observe the disease’s progression. There are three types of microscopic tests that indicate the existence of immuno-complex deposition in the glomeruli, these are light microscopy, electron microscopy, and immunofluorescence [10].

Light Microscopy

The light microscopy shows a diffused proliferative glomerulonephritis with prominent capillaries proliferation and neutrophils. Glomerular involvement is generally uniform and enlarged, glomeruli fill Bowman's space. the severity of the PSGN is related to the findings, patients with the asymptomatic disease will have less glomerular enlargement, and in patients with acute renal failure, the findings are more proliferative capillary with glomerular enlargement [10].

Electron Microscopy and Immunofluorescence

Electron microscopy will show dome-shaped subepithelial deposits. These deposits are called humps. Immunofluorescence microscopy shows a pattern of deposits of immunoglobulin IgG and C3 complement within the mesangium (a cellular network in the renal glomerulus that helps support the capillary loops [10,15]) and glomerular capillaries. The C3 deposition pattern -garland type deposits- give a pattern called starry sky pattern, also another immune reactant may be detected like IgA, IgM, or fibrin [10,15].

Urinalysis

Urinalysis will reveal in PSGN patients hematuria and proteinuria, which are red blood cells and protein in urine respectively. The amount varies in proteinuria, and in hematuria, there are dysmorphic RBCs [1]. Urinalysis must be performed on a freshly voided specimen that is taken after the external urethral area is cleaned with liquid soap and rinsed well [15].

Blood Sample

A blood sample is taken from a PSGN patient to observe the GFR, complement level, and comprehensive metabolic profile. Rising serum creatinine is indicative for reduce glomerular function. Regarding complement level, in 90% in the first two-week presentation, there is a slight decrease in CH50 and C3. In some patients, C4 and C2 levels are low, which suggests that there is an activation of complement in alternative pathways [10]. Lastly, a comprehensive metabolic panel (CMP) is a chemical screening that reveals elevated creatinine and hypoalbuminemia, which indicate advanced disease and nephrotic syndrome respectively [10].

Serology Test

A serology test will reveal elevated titers of extracellular streptococcal antibodies which is evidence of acute infection of GAS13, it is done by stretozyme test, which measures five streptococcal antibodies, they are anti-streptolysin O (ASO), anti-hyaluronidase (AHase), anti-streptokinase (ASKase), anti-nicotinamide-adenin dinucleotidase (anti-NAD) and anti-DNase B antibodies. Patients with PSGN will have high titers of ASO and AHase as well as positive anti-NAD test [10].

Ultrasound and X-ray

The imaging studies in PSGN could be used to assess the possible complications of PSGN such as pulmonary congestion and chronic kidney disease. Imaging modalities such as ultrasound and chest x-ray can be helpful in some cases, yet they are not diagnostic. Renal ultrasound may show renal enlargement in some PSGN patients, while chest x-ray may show evidence of pulmonary congestion in some patients who show symptoms of heart failure and hypervolemia [2,10].

Management of PSGN

PSGN is usually a self-limiting disease that requires symptomatic treatment. The management is supportive, and the goal is to preserve the renal function or to reverse renal damage [2]. In patients with hypervolemia, intravenous furosemide is indicated to relieve volume overload signs and symptoms, which is a loop diuretic that inhibits the reabsorption of Na and Cl ions. It can be used to manage edema and hypertension. In hypertensive patients that require antihypertensive therapy, vasodilators are more effective than antihypertensive. Potassium-sparing diuretics are contradicted due to the tendency to hyperkalemia [2]. Using appropriate antibiotic treatment course in early detected acute PSGN as well as the prophylactic dose is effective in preventing the spread of streptococcal associated glomerulonephritis which are given to groups of high risk, but the problem is the uncertainty with diagnosing streptococcal infection in some cases as the primary cause of infection may occur with different microorganisms. Nevertheless, with the help of some scoring systems in identifying who should be given antibiotic course like the scale of the Centor and McIsaac scores to predict Group A Streptococcal pharyngitis using antibiotic therapy will be beneficial, such treatment is phenoxymethylpenicillin, is indicated for reducing the disease severity and in patients with recurrent GAS infection [2]. Finally, penicillin can be prescribed as prophylactic for GAS carrier state, epidemics, and in those exposed to close contact with cases to prevent the spread of infection to others [16].

Emerging treatments have shown possible management of PSGN like immunoadsorption, which is a blood purification technique used to eliminate pathogenic antibodies. Also, recent experiments concluded that CD28-B7 blockade reduced autoantibody production and cellular infiltration of glomeruli, and prevented target organs, and prevent the development of PSGN [17-19].

Complications of PSGN

Short-Term Complications

PSGN complications are of two types and it depends on the presence of treatment as soon as the symptoms manifest on the patient. The first type is a short-term complication which is when the symptoms first appear, it could cause hypervolemia, which is mainly caused by decreased GFR, and this condition will cause edema and needs a diuretic to return the normal volume. Also, hypertension could develop due to sodium retention; this is also managed with diuretics [10,20].

Long-Term Complications

If PSGN is not treated, this will cause the second type of complication which is a long-term complication, and the patient may develop chronic renal failure. The main determinants of a relatively poor renal outcome include lack of response to initial treatment, more severe proteinuria, more severe renal dysfunction at presentation, and an increased amount of fibrotic changes [10,20].

Prognosis of PSGN

Short-Term Prognosis

Patients with PSGN are least likely to develop chronic kidney disease as they are getting treated for the underlying disease, but most patients, especially children, eventually have complete recovery from the initial episode. In other words, if the underlying infection is treated the patient will have a great prognosis [2,21].

The Resolving of Symptoms

Even though the persistence of elevated pressures for six weeks is compatible with the resolution, the edema usually resolves within five to 10 days and the blood pressure returns to normal after two to three weeks. Urinary abnormalities begin to resolve at various times after onset. Proteinuria will disappear or decrease within the first three months. However, postural or intermittent proteinuria will be present for one to two years after the onset of the disease [12].

Long-Term Prognosis

The long-term prognosis of PSGN may be influenced by the coexistence of other risk factors of chronic renal failure such as metabolic syndrome and diabetes mellitus [9]. Virtually all studies report a good prognosis for the vast majority of children with PSGN [12]. However, patients will PSGN will not always have a benign long-term prognosis [5]. Some of the affected patients, adults particularly, may develop renal failure, hypertension, and recurrent proteinuria [22].

Prevention of PSGN

Prevention of GAS Infection

The main way to prevent PSGN is to prevent group A strep infections like impetigo, strep throat, and scarlet fever. There is no vaccine for GAS infection [23].

Health Promotion

Modern healthcare with the help of the evidence-based medicine (EBM) approach provides special care about the quality of life and the health of the community regarding those most in need, with infection control, sterilization, and hygiene being some of the most influential factors in the health system. Poorer communities have additional health costs, increased patient suffering, and decreased patient compliance. Finally, to reach the maximum results with minimum effort, we must keep our orientation towards maintaining the health community as best as possible [24].

## Conclusions

The most prevalent medical condition which causes acute nephritis world widely is PSGN. It has been obvious that the risk of PSGN is increasing in third world countries and is attributed to many factors including poor hygiene and weak health infrastructure. It is mainly considered the most common cause of acute nephritis worldwide. As mentioned above, children between five and 12 years of age as well as adults over 60 years of age are highly vulnerable to PSGN. Even though the clinical findings of PSGN commonly include hypertension, gross hematuria, and edema, the manifestation of PSGN usually varies from asymptomatic or small microscopic hematuria to more progressively acute nephritic syndrome. The paraclinical findings include wide abnormalities such as abnormal urinalysis (pyuria, red blood cell casts, different grads of proteinuria, and dysmorphic red blood cells), as well as a positive serological test for antibodies to streptococcal antigens, and a low level of complements in the blood (hypocomplementemia).

The main mechanism of glomerular damage is antibody-mediated immune injury and one more thing which is very important to understand is that is commonly by complement-mediated action and leukocyte-mediated pathways such as leukocyte recruitment with the release of various mediators, and sometimes by direct podocyte damage. Cells in the glomerulus may also directly be damaged by antibodies as humoral immunity effect. Furthermore, the formation of the immune complex is the primary landmark of antibody-mediated glomerulonephritis diseases. It is maybe in form of disposition of circulating immune complexes or occurring in situ. These immune complexes are formed in two ways, either contain endogenous antigens (e.g. in membranous nephropathy) or circulating exogenous antigens (e.g. microbial). The deposition of immune complexes appears in a granular pattern. Ultimately, the cause of the anti-GBM antibody-mediated disease is the formation of autoantibodies that attack the components of the GBM which is usually accompanied by severe damage. Antibody deposition is in a sort of linear pattern. Finally, the genetic aspect of PSGN has some features that may responsible for resistance or predisposition to the disease but have not been identified.
